# Cluster analysis of polyphenol intake in a French middle-aged population (aged 35–64 years)

**DOI:** 10.1017/jns.2016.16

**Published:** 2016-07-07

**Authors:** Chantal Julia, Mathilde Touvier, Camille Lassale, Léopold Fezeu, Pilar Galan, Serge Hercberg, Emmanuelle Kesse-Guyot

**Affiliations:** 1 Université Paris 13, Equipe de Recherche en Epidémiologie Nutritionnelle (EREN), Centre de Recherche en Epidémiologie et Statistiques, Inserm (U1153), Inra (U1125), Cnam, COMUE Sorbonne Paris Cité, F-93017 Bobigny, France; 2 Department of Public Health, Avicenne Hospital (AP-HP), Bobigny, France

**Keywords:** Polyphenol intakes, Cluster analysis, Profiles of consumption, MCA, multiple correspondence analysis, SU.VI.MAX, SUpplementation en VItamines et Minéraux AntioXydants

## Abstract

Polyphenols have been suggested as protective factors for a range of chronic diseases. However, studying the impact of individual polyphenols on health is hindered by the intrinsic inter-correlations among polyphenols. Alternatively, studying foods rich in specific polyphenols fails to grasp the ubiquity of these components. Studying overall dietary patterns would allow for a more comprehensive description of polyphenol intakes in the population. Our objective was to identify clusters of dietary polyphenol intakes in a French middle-aged population (35–64 years old). Participants from the primary prevention trial SUpplementation en VItamines et Minéraux AntioXydants (SU.VI.MAX) study were included in the present cross-sectional study (*n* 6092; 57·8 % females; mean age 48·7 (sd 6·4) years). The fifty most consumed individual dietary polyphenols were divided into energy-adjusted tertiles and introduced in a multiple correspondence analysis (MCA), leading to comprehensive factors of dietary polyphenol intakes. The identified factors discriminating polyphenol intakes were used in a hierarchical clustering procedure. Four clusters were identified, corresponding broadly to clustered preferences for their respective food sources. Cluster 1 was characterised by high intakes of tea polyphenols. Cluster 2 was characterised by high intakes of wine polyphenols. Cluster 3 was characterised by high intakes of flavanones and flavones, corresponding to high consumption of fruit and vegetables, and more broadly to a healthier diet. Cluster 4 was characterised by high intakes of hydroxycinnamic acids, but was also associated with alcohol consumption and smoking. Profiles of polyphenol intakes allowed for the identification of meaningful combinations of polyphenol intakes in the diet.

Polyphenols represent a complex family occurring in most plant foods, consisting of more than 500 identified compounds in the human diet, from low-molecular-weight phenolic acids to highly polymerised proanthocyanidins^(^^1^^,^^2^^)^. Bioavailability and biological properties of polyphenols depend on their chemical structure, including the number and position of hydroxyl groups, glycosylation and acylation of the compound^(^^1^^,^^3^^–^^6^^)^. Polyphenols are classified as phenolic acids, flavonoids, stilbenes and lignans according to their chemical structure^(^^7^^)^. Intakes of individual polyphenols or subclasses of polyphenols (particularly flavonoids) have been investigated in various populations and related to their main food sources^(^^8^^–^^17^^)^.

Polyphenols are powerful antioxidants and they have been suggested to be associated with protection against a wide range of chronic diseases^(^^18^^–^^26^^)^. Individual polyphenols or subclasses of polyphenols have shown protective effects in CVD and inflammation^(^^19^^,^^20^^,^^23^^)^. Regarding cancer, however, the most recent reviews have found inconsistent results^(^^18^^,^^22^^,^^24^^)^.

However, studies on the impact of individual polyphenols on health are hindered by the intrinsic intercorrelations among polyphenols sharing common food sources^(^^19^^)^. Some authors have bypassed this limitation by assessing the impact of polyphenol-rich foods (e.g. tea or cocoa) on health^(^^27^^,^^28^^)^. However, studying specific foods is subject to the same kind of limitations, as foods are eaten in combination, within meals^(^^29^^)^. Therefore synergistic or interactive combinations of polyphenols within one food source are likely to also interfere between various foods eaten together^(^^13^^)^. To overcome the limitation of investigating individual polyphenols or foods, some have argued for the investigation of overall dietary patterns^(^^30^^,^^31^^)^.

Studying overall dietary patterns would allow for a more comprehensive description of combinations of individual polyphenols within an individual's diet. Our objective was to identify mutually exclusive groups of dietary polyphenol intakes in a French middle-aged population, in order to identify meaningful combinations of individual polyphenol intakes within the diet.

## Material and methods

### Population

Subjects included in the present study were selected from participants in the SUpplementation en VItamines et Minéraux AntioXydants (SU.VI.MAX) study. Briefly, middle-aged participants from the general population (35–64 years old) were included in 1994–1995 in a randomised, double-blind, placebo-controlled, primary prevention trial (Trial Registration clinicaltrials.gov no. NCT00272428) designed to evaluate the effect of a planned 8-year supplementation in antioxidant vitamins and minerals at nutritional doses on the incidence of CVD and cancer^(^^32^^)^. This study is a cross-sectional observational study using baseline data from the SU.VI.MAX study.

### Ethics

The SU.VI.MAX study was approved by the Ethics Committee for Studies with Human Subjects of Paris-Cochin Hospital (no. 706) and the Commission Nationale Informatique et Liberté (no. 334641). All subjects gave written informed consent to participate in the study.

### Dietary data assessment

Dietary assessment was carried out via repeated 24 h records (1994–1996), collected by computerised questionnaires using the Minitel Telematic Network loaded with study-specific software, as described before^(^^32^^)^. The Minitel was a small terminal widely used in France as an adjunct to the telephone. Dietary collection dates were randomised and fixed for each participant so that each day of the week and all seasons were covered. A validated instruction manual was used to code food portions, including more than 250 generic items, corresponding to 1000 specific foods^(^^33^^)^. Foods were classified into thirty-two food groups. A French published food composition table was used to calculate nutrient intakes from 24 h dietary records^(^^34^^)^. A specific food composition table was used to compute dietary polyphenol intake, based on the published Phenol-Explorer Database (www.phenol-explorer.eu)^(^^1^^)^. The database contains food-composition data for all known polyphenols (flavonoids, phenolic acids, lignans, stilbenes and other minor polyphenols) in foods. Moreover, it includes data on glycosides and esters. It contains data on a total of 502 polyphenols^(^^1^^)^. Individual polyphenols’ contents in foods were determined by chromatography (most often reverse-phase HPLC and gas chromatography), except for proanthocyanidins > 4mers, for which content data obtained by normal-phase HPLC were used.

Subjects having at least six dietary records available in the first 2 years of the study (1994–1996), with at least three records during the autumn–winter months and three during the spring–summer months, were included in the present study. The number of dietary records retained and the balance of dietary records between seasons were chosen in order to take into account day-to-day and seasonal intra-individual variability in food intake.

### Sociodemographic and anthropometric data

Educational level (primary, secondary, superior), physical activity (irregular, <1 h equivalent walking/d, ≥1 h equivalent walking/d) and smoking status, including cigarettes, cigars and pipes (never smoked, former smoker, current smoker) data were obtained through self-administered questionnaires at baseline.

Anthropometric measurements were taken at a clinical examination 1 year after inclusion in the SU.VI.MAX study. Weight was measured in subjects in light clothing and with no shoes to the nearest 0·1 kg and height was measured to the nearest 1 cm with a wall-mounted stadiometer under the same conditions. When measured weight and height were not available, self-reported weight and height were used instead of measured data (*n*  988; 16·2 %).

### Statistical analysis

BMI was calculated as weight (in kg) divided by the square of height (in m).

Adherence to the traditional Mediterranean diet was computed using the Mediterranean Diet Score, as described by Trichopoulou *et al*.^(^^35^^)^.

Mean daily intake of each nutrient and polyphenol was calculated for each subject across their 24 h dietary records. Then, median intakes of individual polyphenols were computed for the whole population, and the fifty most consumed polyphenols (according to median intake) were considered for the subsequent analysis of clusters of polyphenol intakes. The objective of the analysis was to group individuals in mutually exclusive groups according to their overall intakes in the fifty selected polyphenols.

Polyphenol intakes, nutrient intakes and food group consumption were considered in terms of energy-adjusted intakes using the residual method^(^^36^^)^. Energy-adjusted intakes of the fifty selected individual polyphenols were divided in tertiles and then introduced as input variables in a multiple correspondence analysis (MCA). Factors extracted from the MCA were selected for a subsequent cluster analysis. The number of dimensions used for cluster analysis was selected using the explained inertia (% of the initial variability) that they represented. The dimensions were retained in the analyses if they represented >7 % of total inertia and the number of clusters to include in the model was selected using the plot of semi-partial *R*^2^ and the cubic clustering criterion by the number of clusters.

The identified clusters of polyphenol intakes were described in terms of individual polyphenol intakes, sociodemographic, lifestyle and anthropometric data and finally dietary intake (nutrients and food groups). All results for individual polyphenol intakes, nutrient intakes or food group consumption are presented as mean energy-adjusted variables. Clusters were compared using χ^2^ tests for categorical variables and ANOVA for continuous variables, given the normal distribution of the variables, in particular energy-adjusted residuals.

All tests were two-sided and *P* < 0·001 was considered statistically significant, correcting for multiple comparisons. SAS version 9.3 (SAS Institute, Inc.) was used for analyses

## Results

**Fig. 1.**
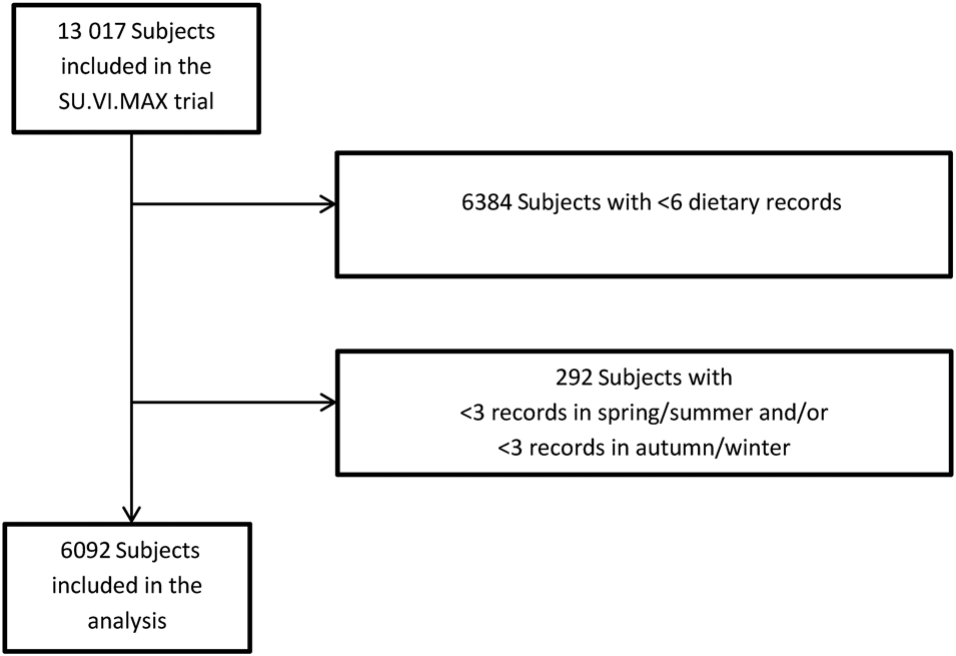
Flowchart of inclusion in the study. SU.VI.MAX, SUpplementation en VItamines et Minéraux AntioXydants.

Among the 13 017 subjects included in the initial SU.VI.MAX study, 6092 had at least six dietary records available (Fig. 1), with at least three in the spring–summer months and three in the autumn–winter months and were included in the study (mean number of dietary records = 11·0  (sd 2·1); mean number of spring–summer dietary records = 5·0  (sd 1·2); mean number of autumn–winter months dietary records = 6·0  (sd 1·6)). The sample included 57·8 % of women, with a mean age of 48·7  (sd 6·4) years.

The fifty most consumed polyphenols belonged mostly to flavonoids and phenolic acids. Selected individual polyphenols included flavanols (six catechins, nine proanthocyanidins (six individual trimers or dimers, three measured using normal-phase HPLC by degree of polymerisation)), one dihydroflavonol, four anthocyanins, three flavanones, three flavones, eight flavonols, two hydroxybenzoic acids, thirteen hydroxycinnamic acids and one other polyphenols (tyrosol). No lignans or stilbens were represented in the selection.

Four main factors were extracted from the MCA procedure, explaining 39 % of total inertia. Plots of the cubic clustering criteria and semi-partial *R*^2^ by the number of clusters allowed us to identify four clusters of dietary polyphenol intakes as the best solution.

The four clusters regrouped 1352 (22·2 %), 1355 (22·2 %), 1456 (23·9 %) and 1929 (31·7 %) subjects, respectively (Tables 1 and 2). Cluster 1 corresponded to higher intakes of individual catechins, proanthocyanidins, hydroxybenzoic acids and some flavonols, and lower intakes of individual hydroxycinnamic acids. Cluster 2 corresponded to higher intakes of malvidin, dihydromyricetin 3-*O*-rhamnoside, quercetin, caffeic acid, caffeoyl tartaric acid and tyrosol and lower intakes of apigenin, quercetin 3,4′-*O*-diglucoside and ferulic acid. Cluster 3 corresponded to higher intakes of the selected flavanones, flavones and lower intakes of individual proanthocyanidins. Cluster 4 corresponded to higher intakes of hydroxycinnaminic acids and lower intakes of flavonols, catechins, flavanones and proanthocyanidins (measured by normal-phase HPLC by degree of polymerisation). Detailed intakes of main classes and subclasses of polyphenols are available in Supplementary Table S3.

**Table 1.** Intake of fifty individual polyphenols for the identified clusters – flavonoid compounds* (Mean values and standard deviations)

	Cluster 1		Cluster 2		Cluster 3		Cluster 4		
	Mean	sd	Mean	sd	Mean	sd	Mean	sd	*P*†
*n*	1352		1355		1456		1929		
%	22·2		22·2		23·9		31·7		
Flavonoids									
Flavanones									
Hesperidin (mg/d)	16·8	19·10	16·7	18·88	17·5‡	21·79	15·8§	19·02	0·08
Narirutin (mg/d)	3·5	3·96	3·4	3·91	3·6‡	4·52	3·3§	3·95	0·08
Didymin (mg/d)	3·8	4·38	3·8	4·33	4·0‡	5·00	3·6§	4·37	0·08
Flavones									
Apigenin 6,8-di-C-glucoside (mg/d)	3·6	4·09	3·6	4·04	3·7‡	4·66	3·4§	4·07	0·08
Apigenin arabinoside-glucoside (mg/d)	10·4	4·32	9·9§	3·84	11·1‡	5·55	10·4	4·72	<0·001
Apigenin galactoside-arabinoside (mg/d)	16·1	7·51	15·2§	6·58	17·3‡	9·64	16·0	8·13	<0·001
Flavonols									
Quercetin (mg/d)	3·5	1·72	3·5‡	1·72	2·4§	2·21	3·1	2·02	<0·001
Quercetin 3-*O*-galactoside (mg/d)	3·6‡	2·58	2·5	2·14	3·1	2·59	2·1§	1·82	<0·001
Quercetin 3-*O*-glucoside (mg/d)	5·1‡	2·97	3·8	2·41	2·4§	2·55	2·5	1·92	<0·001
Quercetin 3-*O*-rhamnoside (mg/d)	3·3‡	1·54	3·3	1·60	1·5§	1·13	2·8	1·75	<0·001
Quercetin 3-*O*-rutinoside (mg/d)	7·6‡	4·93	5·5	3·88	5·0	4·66	4·0§	3·02	<0·001
Quercetin 3,4′-*O*-diglucoside (mg/d)	5·3	3·11	5·2§	3·44	5·7‡	4·01	5·2§	3·50	<0·001
Kaempferol 3-*O*-glucoside (mg/d)	4·3‡	2·92	3·0	2·34	2·2	2·63	1·9§	1·87	<0·001
Quercetin 4′-*O*-glucoside (mg/d)	3·4	2·02	3·4	2·24	3·7‡	2·61	3·4§	2·28	<0·001
Anthocyanins									
Cyanidin 3-*O*-rutinoside (mg/d)	11·9	19·59	10·6	20·37	12·2‡	23·21	10·2§	18·85	0·01
Pelargonidin 3-*O*-glucoside (mg/d)	6·3	7·61	5·7	7·09	6·5‡	8·24	5·6§	7·22	0·002
Malvidin 3-*O*-glucoside (mg/d)	17·7	12·43	18·0‡	12·85	1·8§	6·02	13·1	14·07	<0·001
Malvidin 3-*O*-(6″-acetyl-glucoside) (mg/d)	6·3	4·42	6·4‡	4·61	0·5§	1·86	4·6	5·00	<0·001
Dihydroflavonols									
Dihydromyricetin 3-*O*-rhamnoside (mg/d)	7·5	5·53	7·7‡	5·82	0·2§	1·97	5·5	6·27	<0·001
Flavanols									
Catechins									
(+)-Catechin (mg/d)	22·5‡	9·88	19·6	9·36	10·1§	7·65	14·7§	9·72	<0·001
(−)-Epicatechin (mg/d)	30·9‡	14·95	24·0	12·18	22·4	15·94	19·1§	10·09	<0·001
(+)-Gallocatechin (mg/d)	27·7‡	30·80	12·9	22·43	18·0	27·77	5·0§	12·60	<0·001
(−)-Epigallocatechin (mg/d)	24·7‡	27·43	11·5	19·97	16·1	24·74	4·5§	11·23	<0·001
(−)-Epicatechin 3-*O*-gallate (mg/d)	19·4‡	20·03	9·7	14·62	11·9	18·12	4·2§	8·26	<0·001
(−)-Epigallocatechin 3-*O*-gallate (mg/d)	32·5‡	36·34	15·0	26·47	21·2	32·76	5·7§	14·87	<0·001
Proanthocyanidins, by DP-HPLC									
Proanthocyanidins by DP-HPLC (>10mers) (mg/d)	88·9‡	49·00	81·7	41·08	81·6	78·09	77·5§	44·69	<0·001
Proanthocyanidins by DP-HPLC (4–6mers) (mg/d)	58·4‡	32·17	54·5	28·80	56·0	54·21	52·8§	32·86	<0·001
Proanthocyanidins by DP-HPLC (7–10mers) (mg/d)	39·7‡	21·15	37·1	18·64	36·5	33·52	35·5§	20·79	<0·001
Proanthocyanidins									
Procyanidin dimer B1 (mg/d)	20·6‡	11·51	16·1	10·20	11·6§	11·27	12·0	10·07	<0·001
Procyanidin dimer B2 (mg/d)	25·3‡	10·97	22·2	10·43	17·0§	12·17	19·0	11·19	<0·001
Procyanidin dimer B3 (mg/d)	17·7‡	11·59	17·5	12·23	1·9§	4·28	12·4	13·18	<0·001
Procyanidin dimer B4 (mg/d)	16·7‡	9·80	14·6	9·92	3·3§	5·37	9·7	10·30	<0·001
Procyanidin trimer C1 (mg/d)	8·6‡	4·18	7·3	3·78	4·0§	3·81	5·6	4·17	<0·001
Procyanidin trimer T2 (mg/d)	11·1‡	8·28	11·5	8·73	0·3§	2·93	8·1	9·37	<0·001

DP-HPLC, normal-phase HPLC by degree of polymerisation.

* Mean intakes are calculated from residuals after taking into account energy intake.

† *P* value from mean comparison by ANOVA.

‡ Clusters with the highest mean intake of individual polyphenols.

§ Clusters with the lowest mean intake of individual polyphenols.

**Table 2.** Intake of fifty individual polyphenols for the identified clusters – phenolic acids and other polyphenols* (Mean values and standard deviations; number of subjects and percentages)

	Cluster 1		Cluster 2		Cluster 3		Cluster 4		
	Mean	sd	Mean	sd	Mean	sd	Mean	sd	*P*†
Subjects									
*n*	352		1355		1456		1929		
%	22·2		22·2		23·9		31·7		
Phenolic acids									
Hydroxybenzoic acids									
Gallic acid (mg/d)	18·0‡	12·28	13·4	11·14	8·9	10·99	8·8§	8·38	<0·001
5-*O*-Galloylquinic acid (mg/d)	27·0‡	30·17	12·4	21·98	17·6	27·20	4·8§	12·35	<0·001
Hydroxycinnamic acids									
4-*p*-Coumaroylquinic acid (mg/d)	3·0‡	2·21	2·2	1·88	2·7	2·26	1·8§	1·65	<0·001
Caffeic acid (mg/d)	5·5	2·39	5·6‡	2·50	2·5§	1·37	4·8	2·73	<0·001
Ferulic acid (mg/d)	8·0	2·51	7·9§	2·56	8·5‡	3·47	8·0	2·82	<0·001
Caffeoyl tartaric acid (mg/d)	6·7	4·40	6·9‡	4·49	0·9§	1·85	5·1	4·98	<0·001
3-Caffeoylquinic acid (mg/d)	53·1§	59·55	110·6	26·41	69·2	48·25	249·2‡	97·10	<0·001
4-Caffeoylquinic acid (mg/d)	51·1§	65·94	118·6	27·17	69·5	52·37	276·9‡	110·36	<0·001
5-Caffeoylquinic acid (mg/d)	107·6§	81·29	186·6	39·97	131·6	66·89	374·7‡	133·51	<0·001
5-Feruloylquinic acid (mg/d)	9·4§	12·91	22·9	5·36	13·2	10·37	54·1‡	21·64	<0·001
4-Feruloylquinic acid (mg/d)	6·9§	9·46	16·8	3·93	9·6	7·61	39·6‡	15·86	<0·001
3-Feruloylquinic acid (mg/d)	3·5§	4·62	8·3	1·93	4·9	3·69	19·4‡	7·73	<0·001
3,5-Dicaffeoylquinic acid (mg/d)	1·8§	1·75	3·5	0·80	2·3	1·43	7·7‡	2·90	<0·001
3,4-Dicaffeoylquinic acid (mg/d)	2·5§	2·96	5·6	1·25	3·4	2·37	12·7‡	4·95	<0·001
4,5-Dicaffeoylquinic acid (mg/d)	1·6§	2·26	4·0	0·94	2·3	1·82	9·5‡	3·79	<0·001
Other polyphenols									
Tyrosol (mg/d)	6·1	4·01	6·4‡	4·18	0·8§	1·65	4·7	4·61	<0·001

* Mean intakes are calculated from residuals after taking into account energy intake.

† *P* value from mean comparison by ANOVA.

‡ Clusters with the highest mean intake of individual polyphenols.

§ Clusters with the lowest mean intake of individual polyphenols.

Sociodemographic and lifestyle characteristics of participants according to clusters of dietary polyphenol intakes are shown in Table 3. Compared with other clusters, cluster 1 had the lowest percentage of subjects with no diploma and primary education and had the highest mean age. Cluster 2 had the highest percentage of subjects with irregular physical activity, as well as subjects with the highest BMI. Cluster 3 had the highest percentage of subjects with university education and the lowest percentage of smokers, the lowest mean age and lowest BMI. Cluster 4 had the highest percentage of subjects with no diploma or primary education, the highest percentage of smokers and the highest percentage of subjects with ≥1 h equivalent walking/d.

**Table 3.** Characteristics of the study population by polyphenol cluster (*n* 6092) (Number of subjects and percentages; mean values and standard deviations)

	Cluster 1		Cluster 2		Cluster 3		Cluster 4		
	*n*	%	*n*	%	*n*	%	*n*	%	P*
Subjects	1352	22·2	1355	22·2	1456	23·9	1929	31·7	
Sex									
Men	537	39·7	587	43·3	618	42·4	831	43·1	0·19
Women	815†	60·3	768‡	56·7	838	57·6	1098	56·9	
Educational level									
No diploma and primary	229‡	17·1	291	21·9	282	19·7	443†	23·3	<0·0001
Secondary	551	41·2	522	39·3	538	37·5	718	37·8	
University	557	41·7	516	38·8	614†	42·8	737	38·8	
Smoking status									
Never smoker	678	51·4	585	45·0	846	60·0	730	39·0	<0·0001
Former smoker	488	37·0	548	42·1	466	33·0	785	42·0	
Current smoker	154	11·7	168	12·9	98‡	7·0	355†	19·0	
Physical activity									
Irregular	326	24·4	350†	26·5	319‡	22·4	483	25·4	0·01
<1 h walking/d	429	32·1	418	31·7	476†	33·4	540‡	28·4	
≥1 h walking/d	582	43·5	552‡	41·8	631	44·2	877†	46·2	
Age (years)									<0·0001
Mean	49·5†		49·4		47·9‡		48·4		
sd	6·37		6·16		6·64		6·28		
BMI (kg/m^2^)									<0·0001
Mean	23·8		24·3†		23·6‡		24·2		
sd	3·50		3·61		3·64		3·62		

* *P* from mean comparison by ANOVA.

† Clusters with the highest mean intake of individual polyphenols.

‡ Clusters with the lowest mean intake of individual polyphenols.

Consumption of food groups across clusters is shown in Table 4. Cluster 1 was characterised by very high consumption of tea and low consumption of coffee, sweetened beverages, starchy foods (pasta, rice, potatoes), meat, processed meat and snacks (sweet and savoury). Cluster 2 was characterised by a high consumption of wine, and low consumption of bread and legumes, dairy products, fruit and vegetables, fish and low Mediterranean Diet Scores. Cluster 3 was characterised by high consumption of almost all food groups, but more importantly of fruit, vegetables, fish, milk and dairy products and starchy foods, but also sweetened beverages and snacks. It was also characterised by a high Mediterranean Diet Score. It also had the lowest consumption of wine. Cluster 4 was characterised by high consumption of coffee, spirits and beer, meat and processed meat, and low consumption of tea, milk and breakfast cereals.

**Table 4.** Food group intake (g/d) by polyphenol cluster (*n* 6092) (Mean values and standard deviations)

	Cluster 1		Cluster 2		Cluster 3		Cluster 4		
	Mean	sd	Mean	sd	Mean	sd	Mean	sd	P*
Coffee	74·4‡	110·47	191·6	51·13	119·5	90·82	462·6†	185·74	<0·001
Tea	260·6†	286·03	121·7	207·76	156·8	258·84	43·3‡	111·73	<0·001
Spirits	18·7	36·43	21·8	34·27	15·0	32·14	23·2†	45·91	<0·001
Beer and cider	29·4	62·94	36·9	78·89	33·5	91·40	39·7†	83·56	0·002
Wine	194·5	191·74	208·6†	190·73	61·3‡	93·72	168·6	188·12	<0·001
Sweetened beverages	14·0‡	32·40	14·5	36·85	23·5†	58·73	19·4	47·80	<0·001
Fruit juice	40·8	61·49	39·3‡	61·32	43·8†	62·35	39·5	59·71	0·16
Vegetables juice	1·0	7·33	0·8	5·06	1·3	10·44	1·1	10·13	0·40
Breakfast cereals	6·5	13·23	5·7	13·57	8·1†	16·64	4·9‡	11·84	<0·001
Flour	3·2	2·64	2·9‡	2·53	3·9†	3·26	3·2	2·83	<0·001
Pasta, rice	52·8‡	38·23	54·3	40·21	69·0†	52·55	58·1	41·30	<0·001
Potatoes	65·4‡	44·93	68·0	45·94	84·0†	60·41	76·5	55·93	<0·001
Bread	110·1	60·77	109·2‡	63·13	147·0†	70·98	123·9	66·37	<0·001
Legumes	10·7	15·41	10·4‡	15·38	13·6†	18·74	11·5	16·82	<0·001
Milk	110·4	129·94	135·9	127·93	197·4†	162·58	96·1‡	131·31	<0·001
Yogurt	73·6	64·79	70·0‡	64·51	80·4†	67·79	76·4	72·78	0·001
Cream cheese	18·3	31·38	16·7‡	28·18	20·7†	33·99	19·3	32·32	0·006
Cheese	42·3‡	27·64	43·1	28·50	50·1†	32·12	48·6	33·09	<0·001
Fruit	182·7	111·82	172·1‡	107·99	216·8†	131·86	184·1	120·62	<0·001
Dried fruit	3·7	5·77	3·0‡	4·17	4·6†	6·48	3·3	5·37	<0·001
Vegetables	200·5	77·11	198·5‡	80·79	226·4†	90·20	208·8	87·84	<0·001
Animal fat	17·7	10·37	17·3‡	10·55	23·5†	12·64	20·6	11·95	<0·001
Vegetable fat	18·3‡	9·23	18·3‡	9·00	21·0†	10·19	19·2	9·41	<0·001
Fish	34·8	25·82	32·6‡	25·01	35·7†	27·74	32·8	26·06	0·002
Seafood	11·0	14·49	10·8	13·86	9·8	15·00	10·7	14·69	0·1
Meat	112·1‡	47·00	118·1	48·85	120·9	49·54	125·1†	50·63	<0·001
Processed meat	25·2‡	20·11	27·5	22·31	29·8†	23·25	29·8†	22·32	<0·001
Salty snacks	4·7‡	4·42	5·3	5·30	6·1†	6·16	5·6	5·59	<0·001
Sweets and snacks	80·1‡	46·96	84·7	47·79	115·5†	55·83	95·3	54·25	<0·001
Mediterranean Diet Score	4·53	1·57	4·27	1·60	4·56	1·57	4·53	1·52	<0·001

* *P* value from mean comparison by ANOVA.

† Clusters with the highest mean intake of individual polyphenols.

‡ Clusters with the lowest mean intake of individual polyphenols.

Nutrient intakes across clusters are shown in Table 5. Cluster 1 had the lowest energy intake, and the lowest intakes of saturated and polyunsaturated fat, as well as lowest intakes of vitamins E and B_9_, Ca, Na and Fe. Cluster 2 had the lowest energy intake from carbohydrates, highest energy intake from proteins and alcohol; cluster 2 had the lowest intake of dietary fibres, β-carotene, vitamin C and vitamin D. Cluster 3 had the highest energy intake, highest energy intake from carbohydrates and fat, and the lowest energy intake from proteins and alcohol and the highest intakes of all nutrients: fibres, saturated and polyunsaturated fat, vitamins and trace elements. Participants in cluster 4 did not have particularly high or low nutrient intakes compared with other clusters.

**Table 5.** Nutrient intake by polyphenol cluster (*n* 6092) (Mean values and standard deviations)

	Cluster 1		Cluster 2		Cluster 3		Cluster 4		
	Mean	sd	Mean	sd	Mean	sd	Mean	sd	P*
Energy intake									<0·001
kJ	8210·3‡	2462·16	8427·4	2566·80	9824·0†	2064·22	8976·4	2428·64	
kcal	1962·3‡	588·47	2014·2	613·48	2348·0†	493·36	2145·4	580·46	
% Energy from carbohydrates	38·7	6·23	37·8‡	6·03	42·3†	5·78	38·7	6·60	<0·001
% Energy from fat	37·5‡	5·02	37·7	4·91	38·9†	4·71	38·3	4·86	<0·001
% Energy from proteins	16·8	2·65	16·9†	2·67	16·3‡	2·23	16·7	2·50	<0·001
% Energy from alcohol	7·0	5·76	7·6†	5·76	2·4‡	3·21	6·3	6·15	<0·001
Total dietary fibres (g/d)	17·9	5·78	17·5‡	5·92	22·2†	6·85	18·8	6·39	<0·001
Saturated fat (g/d)	32·9‡	10·67	34·3	11·46	42·3†	10·96	37·7	11·98	<0·001
Polyunsaturated fat (g/d)	12·3‡	4·43	12·5	4·48	14·8†	4·38	13·4	4·53	<0·001
β-Carotene (μg/d)	3720·2	2027·87	3639·1‡	1924·13	4434·3†	2597·09	3930·7	2323·93	<0·001
Vitamin C (mg/d)	92·7	43·16	90·5‡	43·44	104·7†	44·57	92·5	42·81	<0·001
Vitamin E (mg/d)	11·9‡	4·28	12·0	4·37	14·3†	4·73	12·8	4·50	<0·001
Vitamin B_9_ (μg/d)	295·4‡	88·60	295·9	94·04	347·1†	92·00	308·6	92·33	<0·001
Vitamin B_12_ (μg/d)	6·8‡	4·10	7·2	4·45	7·4†	3·89	7·1	3·95	<0·001
Ca (mg/d)	879·3‡	289·75	912·3	288·07	1103·1†	320·93	932·7	319·79	<0·001
Vitamin D (μg/d)	2·8	1·82	2·7‡	1·67	2·9†	1·87	2·9	1·76	<0·001
Na (mg/d)	3178·0‡	1071·45	3239·9	1133·86	3867·4†	1122·16	3487·5	1157·21	<0·001
Fe (mg/d)	12·4‡	4·27	12·6	4·27	13·2†	3·41	12·7	3·90	<0·001

* *P* value from mean comparison by ANOVA.

† Clusters with the highest mean intake of individual polyphenols.

‡ Clusters with the lowest mean intake of individual polyphenols.

## Discussion

Using detailed dietary data from a large sample from the general population, we were able to identify specific profiles of polyphenol intake. Given the fact that most polyphenol compounds share food sources, ascertaining independent effects of dietary components is often subject to multicollinearity and therefore challenging. Thus, it is difficult to disentangle the potential effect of each individual polyphenol and of overall dietary polyphenols from the effect of other food constituents (such as antioxidant vitamins and minerals) provided by the same vector food.

Our results show that individual polyphenols from a single subclass can be associated with different patterns of intakes (for example, individual flavonols are associated with three different clusters). However, to date, polyphenol intakes have been mostly investigated as classes and subclasses (flavonoids, anthocyanins or ‘coffee polyphenols’), focusing on major contributing foods or relating their intake to specific health outcomes^(^^7^^,^^9^^–^^14^^,^^37^^,^^38^^)^. In the light of our results, such an approach could lead some elements of differing dietary behaviours to be integrated into a single indicator. Our study shows that taking into account dietary patterns of polyphenols is crucial, given the observed associations of polyphenol intakes within individuals’ diets. To our knowledge, this is the first study investigating clusters of polyphenol intakes in a general population sample. A holistic approach combining polyphenol intakes in a single *a priori* score has been recently developed, and has been shown to be associated with the Mediterranean diet and low-grade inflammation^(^^39^^,^^40^^)^. Such an approach is complementary to *a posteriori* patterns, as it builds on current knowledge on the relationships between polyphenol intakes and health, while our approach aimed at investigating natural occurring associations of dietary polyphenols within the population's diets.

Interestingly, tea appeared as a major discriminant factor for cluster identification. Polyphenols contained in tea include catechins (tea contributing from 15 to 63 % of intake), procyanidin dimers and trimers (tea contributing from 12 to 48 % of intake), flavonols (kaempferol and quercetin compounds, tea contributing from 3 to 56 % of intake) and theaflavins (tea contributing to 100 % of their intake). However, only four catechins out of the fifty selected individual polyphenols for analyses had tea as their main food source; for these five, tea contributed to about 55 % of intake for each (see Supplementary Tables S1 and S2). Besides, cluster 1 was characterised by high levels of other polyphenol intakes (proanthocyanidins and quercetin compounds) for which tea appeared but as a minor contributor. Conversely, specific flavonoid compounds of tea, theaflavins, which are obtained through tea leaf processing, were not consumed in the population in sufficiently high amounts to be included in the individual polyphenols selected for the MCA procedure^(^^41^^)^. Tea is one of the major sources of polyphenols in Western diets^(^^7^^,^^9^^,^^12^^,^^37^^)^. In a study using a sample of subjects from the National Health and Nutrition Examination Survey (NHANES), 21·3 % of the population consumed tea^(^^37^^)^, yet tea was the major polyphenol source for the whole sample^(^^9^^)^. Consistent with our results, tea consumers had higher intakes of flavonols and catechins^(^^37^^)^. Moreover, sociodemographic profiles identified in the NHANES were similar to those from our study, as tea consumers were more likely to be women and older; however, they also tended to have lower levels of physical activity, which was not observed in our cluster 1^(^^37^^)^. Similarly, in the European Prospective Investigation into Cancer and Nutrition (EPIC) study, determinants of intake of theaflavins were higher diplomas and lower BMI^(^^11^^)^.

Opposing consumptions were observed between tea and coffee. Coffee and tea compete with each other at a world level, countries having a preference for either one beverage or the other^(^^42^^)^. However, competing patterns of consumption have not always been identified at the individual level. Frary *et al*. analysed patterns of beverage consumption in the USA, and among the six identified clusters, one included consumers of both tea and coffee^(^^43^^)^. However, although the USA shares Europe's preference for coffee over tea^(^^42^^)^, overall beverage consumption patterns differ^(^^44^^)^.

Cluster 4 was characterised by high intakes of coffee, beer and spirits associated with smoking. Paired associations between alcohol and tobacco have long been identified^(^^45^^)^, as well as a clustering of risky behaviours in smokers: smokers tend to have higher intakes of alcohol, unhealthier diets and lower levels of physical activity^(^^46^^,^^47^^)^. Paired associations have also been observed between coffee intake and smoking, and alcohol consumption and coffee intake^(^^45^^,^^48^^)^. Consistent with our results, associations were observed in the French prospective cohort study E3N-EPIC (Etude Epidémiologique auprès des femmes de la Mutuelle Générale de l'Education Nationale – European Prospective Investigation into Cancer and Nutrition) between alcohol intake, smoking status and consumption of coffee^(^^49^^)^. Moreover, as in cluster 4, alcohol consumption was associated with intakes of processed meat^(^^49^^)^. This clustered association between coffee, smoking and alcohol, could in part explain the conflicting results observed in the associations between coffee intake and CVD^(^^50^^,^^51^^)^.

Cluster 2 was characterised by high consumption of wine, intermediate consumption of both coffee and tea and low consumption of other food groups. Cluster 3 was characterised by high consumption in almost all food groups considered. Our results confirm that results from single sources of polyphenols, such as tea, coffee or wine, should be considered with caution, given the high level of correlations observed in intakes of these polyphenols. Approaching these correlations through the identification of profiles of intake should allow for complementary information as to the association between polyphenols and mortality and health events, taking into account interactions and confounding with sociodemographic factors (sex in particular).

The most consumed polyphenols identified in our sample were in accordance with previous results from the same population^(^^13^^)^. In the EPIC study, which used also in part data from the Phenol-Explorer Database, intakes of catechins in the French sample were somewhat lower, but comparable with intakes in our study^(^^11^^)^. Comparison with other populations is, however, difficult, due to the heterogeneity in the food composition data used (for example, in the Finnish cohort, only aglycone compounds were considered in the analyses)^(^^12^^)^.

Strengths of our study include the use of very detailed and validated dietary information, from repeated 24 h dietary records. Seasonality in intakes was taken into account, through the balanced number of dietary records available for each subject. Moreover, we used comprehensive data from the Phenol-Explorer Database, which builds on current scientific literature to expand data on individual polyphenol content of foods^(^^2^^)^.

The present study is subject to limitations. Dietary assessment is based on self-reported data, and therefore subject to some subjectivity on portion size estimation or subject to desirability or memory bias. Polyphenol content of foods can vary considerably depending on various factors such as the degree of ripeness of fruits and vegetables, the degree of sunlight exposure, storage conditions or culinary methods which could not be assessed in our sample^(^^7^^)^. Moreover, the Phenol-Explorer Database is based on scientific publications on the content in polyphenols of foods in the English language, which could lead to a selection bias of data excluding some from European countries. Finally, our sample population was selected from a study including middle-aged participants that started in 1994–1996. Profiles of polyphenol intakes could have changed in the general population since then. Moreover, the analyses were conducted on a sample of the total population in the cohort, thus limiting the representativeness of our analyses. Repeated analyses on a different sample population could allow us to confirm our results.

### Conclusion

Patterns of polyphenol intakes were identified from a large sample from the general population. Future studies should investigate the association between such patterns and subsequent health outcomes, in order to identify meaningful combinations of polyphenols within the diet.

## Supplementary material

For supplementary material accompanying this paper visit http://dx.doi.org/10.1017/jns.2016.16. click here to view supplementary material

## References

[1] Perez-Jimenez J, Neveu V, Vos F, (2010) Systematic analysis of the content of 502 polyphenols in 452 foods and beverages: an application of the Phenol-Explorer Database. J Agric Food Chem 58, 4959–4969. 20302342 10.1021/jf100128b

[2] Neveu V, Perez-Jimenez J, Vos F, (2010) Phenol-Explorer: an online comprehensive database on polyphenol contents in foods. Database (Oxford) 2010, bap024. 10.1093/database/bap024 PMC2860900 20428313

[3] Manach C, Williamson G, Morand C, (2005) Bioavailability and bioefficacy of polyphenols in humans. I. Review of 97 bioavailability studies. Am J Clin Nutr 81, Suppl., 230S–242S. 15640486 10.1093/ajcn/81.1.230S

[4] Loke WM, Proudfoot JM, Stewart S, (2008) Metabolic transformation has a profound effect on anti-inflammatory activity of flavonoids such as quercetin: lack of association between antioxidant and lipoxygenase inhibitory activity. Biochem Pharmacol 75, 1045–1053. 18096136 10.1016/j.bcp.2007.11.002

[5] Lafay S, Morand C, Manach C, (2006) Absorption and metabolism of caffeic acid and chlorogenic acid in the small intestine of rats. Br J Nutr 96, 39–46. 16869989 10.1079/bjn20061714

[6] Nielsen ILF, Chee WSS, Poulsen L, (2006) Bioavailability is improved by enzymatic modification of the citrus flavonoid hesperidin in humans: a randomized, double-blind, crossover trial. J Nutr 136, 404–408. 16424119 10.1093/jn/136.2.404

[7] Manach C, Scalbert A, Morand C, (2004) Polyphenols: food sources and bioavailability. Am J Clin Nutr 79, 727–747. 15113710 10.1093/ajcn/79.5.727

[8] Brat P, George S, Bellamy A, (2006) Daily polyphenol intake in France from fruit and vegetables. J Nutr 136, 2368–2373. 16920856 10.1093/jn/136.9.2368

[9] Chun OK, Chung SJ & Song WO (2007) Estimated dietary flavonoid intake and major food sources of US adults. J Nutr 137, 1244–1252. 17449588 10.1093/jn/137.5.1244

[10] Dilis V & Trichopoulou A (2010) Antioxidant intakes and food sources in Greek adults. J Nutr 140, 1274–1279. 20463143 10.3945/jn.110.121848

[11] Knaze V, Zamora-Ros R, Lujan-Barroso L, (2012) Intake estimation of total and individual flavan-3-ols, proanthocyanidins and theaflavins, their food sources and determinants in the European Prospective Investigation into Cancer and Nutrition (EPIC) study. Br J Nutr 108, 1095–1108. 22186699 10.1017/S0007114511006386

[12] Ovaskainen ML, Torronen R, Koponen JM, (2008) Dietary intake and major food sources of polyphenols in Finnish adults. J Nutr 138, 562–566. 18287367 10.1093/jn/138.3.562

[13] Perez-Jimenez J, Fezeu L, Touvier M, (2011) Dietary intake of 337 polyphenols in French adults. Am J Clin Nutr 93, 1220–1228. 21490142 10.3945/ajcn.110.007096

[14] Wang Y, Chung SJ, Song WO, (2011) Estimation of daily proanthocyanidin intake and major food sources in the U.S. Diet. J Nutr 141, 447–452. 21270367 10.3945/jn.110.133900

[15] Zamora-Ros R, Knaze V, Lujan-Barroso L, (2011) Estimated dietary intakes of flavonols, flavanones and flavones in the European Prospective Investigation into Cancer and Nutrition (EPIC) 24 hour dietary recall cohort. Br J Nutr 106, 1915–1925. 21679483 10.1017/S000711451100239X

[16] Zamora-Ros R, Andres-Lacueva C, Lamuela-Raventos RM, (2010) Estimation of dietary sources and flavonoid intake in a Spanish adult population (EPIC-Spain). J Am Diet Assoc 110, 390–398. 20184989 10.1016/j.jada.2009.11.024

[17] Zamora-Ros R, Knaze V, Lujan-Barroso L, (2011) Estimation of the intake of anthocyanidins and their food sources in the European Prospective Investigation into Cancer and Nutrition (EPIC) study. Br J Nutr 106, 1090–1099. 21481290 10.1017/S0007114511001437

[18] Arts ICW & Hollman PCH (2005) Polyphenols and disease risk in epidemiologic studies. Am J Clin Nutr 81, Suppl., 317S–325S. 15640497 10.1093/ajcn/81.1.317S

[19] Erdman JW, Balentine D, Arab L, (2007) Flavonoids and heart health: proceedings of the ILSI North America Flavonoids Workshop, May 31–June 1, 2005, Washington, DC. J Nutr 137, Suppl., 718S–737S. 17311968 10.1093/jn/137.3.718S

[20] Gonzalez R, Ballester I, Lopez-Posadas R, (2011) Effects of flavonoids and other polyphenols on inflammation. Crit Rev Food Sci Nutr 51, 331–362. 21432698 10.1080/10408390903584094

[21] Knekt P, Kumpulainen J, Jarvinen R, (2002) Flavonoid intake and risk of chronic diseases. Am J Clin Nutr 76, 560–568. 12198000 10.1093/ajcn/76.3.560

[22] Scalbert A, Manach C, Morand C, (2005) Dietary polyphenols and the prevention of diseases. Crit Rev Food Sci Nutr 45, 287–306. 16047496 10.1080/1040869059096

[23] Tangney CC & Rasmussen HE (2013) Polyphenols, inflammation, and cardiovascular disease. Curr Atheroscler Rep 15, 324. 23512608 10.1007/s11883-013-0324-x PMC3651847

[24] Chen AY & Chen YC (2013) A review of the dietary flavonoid, kaempferol on human health and cancer chemoprevention. Food Chem 138, 2099–2107. 23497863 10.1016/j.foodchem.2012.11.139 PMC3601579

[25] Kesse-Guyot E, Fezeu L, Andreeva VA, (2012) Total and specific polyphenol intakes in midlife are associated with cognitive function measured 13 years later. J Nutr 142, 76–83. 22090468 10.3945/jn.111.144428

[26] Touvier M, Druesne-Pecollo N, Kesse-Guyot E, (2013) Dual association between polyphenol intake and breast cancer risk according to alcohol consumption level: a prospective cohort study. Breast Cancer Res Treat 137, 225–236. 23132534 10.1007/s10549-012-2323-y

[27] Peters U, Poole C & Arab L (2001) Does tea affect cardiovascular disease? A meta-analysis. Am J Epidemiol 154, 495–503. 11549554 10.1093/aje/154.6.495

[28] Rimbach G, Melchin M, Moehring J, (2009) Polyphenols from cocoa and vascular health – a critical review. Int J Mol Sci 10, 4290–4309. 20057946 10.3390/ijms10104290 PMC2790109

[29] Jacobs DR, Gross MD & Tapsell LC (2009) Food synergy: an operational concept for understanding nutrition. Am J Clin Nutr 89, S1543–S1548. 10.3945/ajcn.2009.26736B PMC2731586 19279083

[30] Hu FB (2002) Dietary pattern analysis: a new direction in nutritional epidemiology. Curr Opin Lipidol 13, 3–9. 11790957 10.1097/00041433-200202000-00002

[31] Moeller SM, Reedy J, Millen AE, (2007) Dietary patterns: challenges and opportunities in dietary patterns research an Experimental Biology workshop, April 1, 2006. J Am Diet Assoc 107, 1233–1239. 17604756 10.1016/j.jada.2007.03.014

[32] Hercberg S, Galan P, Preziosi P, (2004) The SU.VI.MAX Study: a randomized, placebo-controlled trial of the health effects of antioxidant vitamins and minerals. Arch Intern Med 164, 2335–2342. 15557412 10.1001/archinte.164.21.2335

[33] Le Moullec N, Deheeger M, Preziosi P, (1996) Validation du manuel photos utilisé pour l'enquête alimentaire de l’étude SU.VI.MAX (Validation of the photo manual used for the collection of dietary data in the food survey SU.VI.MAX). Cah Nutr Diet 31, 158–164.

[34] Hercberg Sc (2005) Table de composition SU.VI.MAX des aliments (Food Composition Table for SU.VI.MAX). Paris: INSERM/Economica.

[35] Trichopoulou A, Costacou T, Bamia C, (2003) Adherence to a Mediterranean diet and survival in a Greek population. N Engl J Med 348, 2599–2608. 12826634 10.1056/NEJMoa025039

[36] Willett W & Stampfer MJ (1986) Total energy intake: implications for epidemiologic analyses. Am J Epidemiol 124, 17–27. 3521261 10.1093/oxfordjournals.aje.a114366

[37] Song WO & Chun OK (2008) Tea is the major source of flavan-3-ol and flavonol in the US diet. J Nutr 138, 1543–1547. 10.1093/jn/138.8.1543S 18641204

[38] Tresserra-Rimbau A, Medina-Remon A, Perez-Jimenez J, (2013) Dietary intake and major food sources of polyphenols in a Spanish population at high cardiovascular risk: the PREDIMED study. Nutr Metab Cardiovasc Dis 23, 953–959. 23332727 10.1016/j.numecd.2012.10.008

[39] Pounis G, Di CA, Bonaccio M, (2016) Flavonoid and lignan intake in a Mediterranean population: proposal for a holistic approach in polyphenol dietary analysis, the Moli-sani Study. Eur J Clin Nutr 70, 338–345. 26530928 10.1038/ejcn.2015.178

[40] Pounis G, Bonaccio M, Di CA, (2016) Polyphenol intake is associated with low-grade inflammation, using a novel data analysis from the Moli-sani study. Thromb Haemost 115, 344–352. 26355794 10.1160/TH15-06-0487

[41] Subramanian N, Venkatesh P, Ganguli S, (1999) Role of polyphenol oxidase and peroxidase in the generation of black tea theaflavins. J Agric Food Chem 47, 2571–2578. 10552528 10.1021/jf981042y

[42] Grigg D (2002) The worlds of tea and coffee: patterns of consumption. GeoJournal 57, 283–294.

[43] Frary CD, Johnson RK & Wang MQ (2005) Food sources and intakes of caffeine in the diets of persons in the United States. J Am Diet Assoc 105, 110–113. 15635355 10.1016/j.jada.2004.10.027

[44] Drewnowski A, Rehm CD & Constant F (2013) Water and beverage consumption among adults in the United States: cross-sectional study using data from NHANES 2005–2010. BMC Public Health 13, 1068. 24219567 10.1186/1471-2458-13-1068 PMC3840570

[45] Istvan J & Matarazzo JD (1984) Tobacco, alcohol, and caffeine use – a review of their interrelationships. Psychol Bull 95, 301–326. 6544436

[46] Chiolero A, Wietlisbach V, Ruffieux C, (2006) Clustering of risk behaviors with cigarette consumption: a population-based survey. Prev Med 42, 348–353. 16504277 10.1016/j.ypmed.2006.01.011

[47] Dallongeville J, Marecaux N, Fruchart JC, (1998) Cigarette smoking is associated with unhealthy patterns of nutrient intake: a meta-analysis. J Nutr 128, 1450–1457. 9732304 10.1093/jn/128.9.1450

[48] Tavani A, Bertuzzi M, Negri E, (2001) Alcohol, smoking, coffee and risk of non-fatal acute myocardial infarction in Italy. Eur J Epidemiol 17, 1131–1137. 12530773 10.1023/a:1021276932160

[49] Kesse E, Clavel-Chapelon F, Slimani N, (2001) Do eating habits differ according to alcohol consumption? Results of a study of the French cohort of the European Prospective Investigation into Cancer and Nutrition (E3N-EPIC). Am J Clin Nutr 74, 322–327. 11522555 10.1093/ajcn/74.3.322

[50] Bonita JS, Mandarano M, Shuta D, (2007) Coffee and cardiovascular disease: *in vitro*, cellular, animal, and human studies. Pharmacol Res 55, 187–198. 17368041 10.1016/j.phrs.2007.01.006

[51] Cornelis MC & El-Sohemy A (2007) Coffee, caffeine, and coronary heart disease. Curr Opin Lipidol 18, 13–19. 17218826 10.1097/MOL.0b013e3280127b04

