# Supplementary material for: Cluster analysis of polyphenol intake in a French middle-aged population (aged 35–64 years)
Source: J Nutr Sci. 2016 Jul 7;5:e28. doi: 10.1017/jns.2016.16 (PMC4976116; doi:10.1017/jns.2016.16)
Supplement: Supplementary file 1 [file S2048679016000161sup001.doc]

Supplementary Table S1. Major dietary sources and cluster of consumption for the 50 individual polyphenols – flavonoid compounds

|  | Major sources | Cluster of consumption |
| --- | --- | --- |
| ***Flavonoids*** |  |  |
| **Flavanones** |  |  |
| Hesperidin (mg/j) | Fruit (52%), Fruit juice (40%) | Cluster 3 |
| Narirutin (mg/j) | Fruit (54%), Fruit juice (39%) | Cluster 3 |
| Didymin (mg/j) | Fruit (55%), Fruit juice (37%) | Cluster 3 |
| **Flavones** |  |  |
| Apigenin 6,8-di-C-glucoside (mg/j) | Fruit (55%), Fruit juice (37%) | Cluster 3 |
| Apigenin arabinoside-glucoside (mg/j) | Bread (83%), cakes and pastries (9%) | Cluster 3 |
| Apigenin galactoside-arabinoside (mg/j) | Bread (84%), cakes and pastries (9%) | Cluster 3 |
| **Flavonols** |  |  |
| Quercetin (mg/j) | Cakes and pastries (31%), wine (29%), fruit (21%) | Cluster 2 |
| Quercetin 3-O-galactoside (mg/j) | Fruit (69%), tea (26%) | Cluster 1 |
| Quercetin 3-O-glucoside (mg/j) | Wine (36%), tea (26%), fruit (23%) | Cluster 1 |
| Quercetin 3-O-rhamnoside (mg/j) | Wine (37%), fruit (35%), vegetables (22%) | Cluster 1 |
| Quercetin 3-O-rutinoside (mg/j) | Vegetables (33%), tea (25%), wine (21%) | Cluster 1 |
| Quercetin 3,4'-O-diglucoside (mg/j) | Vegetables (99%) | Cluster 3 |
| Kaempferol 3-O-glucoside (mg/j) | Wine (37%), tea (32%), vegetables (11%) | Cluster 1 |
| Quercetin 4'-O-glucoside (mg/j) | Vegetables (99%) | Cluster 3 |
| **Anthocyanins** |  |  |
| Cyanidin 3-O-rutinoside (mg/j) | Fruit (61%), salty snacks (14%), yogurt (12%) | Cluster 3 |
| Pelargonidin 3-O-glucoside (mg/j) | Fruit (77%), cakes and pastries (15%) | Cluster 3 |
| Malvidin 3-O-glucoside (mg/j) | Wine (73%), fruit (20%) | Cluster 2 |
| Malvidin 3-O-(6''-acetyl-glucoside) (mg/j) | Wine (79%), fruit (20%) | Cluster 2 |
| **Dihydroflavonols** |  |  |
| Dihydromyricetin 3-O-rhamnoside (mg/j) | Wine (97%) | Cluster 2 |
| **Flavanols** |  |  |
| Catechins |  |  |
| (+)-Catechin (mg/j) | Wine (39%), fruit (30%), tea (15%), cakes and pastries (12%) | Cluster 1 |
| (-)-Epicatechin (mg/j) | Fruit (39%), wine (21%), tea (19%), cakes and pastries (18%) | Cluster 1 |
| (+)-Gallocatechin (mg/j) | Tea (54%), wine (33%) | Cluster 1 |
| (-)-Epigallocatechin (mg/j) | Tea (53%), wine (23%) | Cluster 1 |
| (-)-Epicatechin 3-O-gallate (mg/j) | Tea (48%), wine (36%), fruit (14%) | Cluster 1 |
| (-)-Epigallocatechin 3-O-gallate (mg/j) | Tea (57%), cakes and pastries (14%), fruit (13%) | Cluster 1 |
| Proanthocyanidins, by DP-HPLC (mg/j) |  |  |
| Proanthocyanidins by DP-HPLC (>10 mers) (mg/j) | Fruit (51%), cakes and pastries (31%), wine (17%) | Cluster 1 |
| Proanthocyanidins by DP-HPLC (04-06 mers) (mg/j) | Fruit (48%), cakes and pastries (35%), wine (16%) | Cluster 1 |
| Proanthocyanidins by DP-HPLC (07-10 mers) (mg/j) | Fruit (54%), cakes and pastries (27%), wine (17%) | Cluster 1 |
| Proanthocyanidins |  |  |
| Procyanidin dimer B1 (mg/j) | Fruit (34%), wine (33%), tea (22%) | Cluster 1 |
| Procyanidin dimer B2 (mg/j) | Fruit (48%), wine (26%), tea (13%), cakes and pastries (12%) | Cluster 1 |
| Procyanidin dimer B3 (mg/j) | Wine (63%), fruit (16%), tea (12%) | Cluster 1 |
| Procyanidin dimer B4 (mg/j) | Wine (60%), tea (26%), fruit (10%) | Cluster 1 |
| Procyanidin trimer C1 (mg/j) | Wine (39%), cakes and pastries (24%), fruit (18%), tea (17%) | Cluster 1 |
| Procyanidin trimer T2 (mg/j) | Wine (99%) | Cluster 1 |

Supplementary Table S2. Major dietary sources and cluster of consumption for the 50 individual polyphenols – phenolic acids and other polyphenol compounds

|  | Major sources | Cluster of consumption |
| --- | --- | --- |
| ***Phenolic acids*** |  |  |
| **Hydroxybenzoic acids** |  |  |
| Gallic acid (mg/j) | Wine (41%), tea (29%), vegetables (20%) | Cluster 1 |
| 5-O-Galloylquinic acid (mg/j) | Tea (56%), fruit (32%) | Cluster 1 |
| **Hydroxycinnamic acids** |  |  |
| 4-p-Coumaroylquinic | Fruit (71%), tea (26%) | Cluster 1 |
| Caffeic acid (mg/j) | Wine (36%), potatoes (34%), fruit (12%), vegetables (11%) | Cluster 2 |
| Ferulic acid (mg/j) | Bread (53%), cakes and pastries (18%) | Cluster 3 |
| Caffeoyl tartaric acid (mg/j) | Wine (71%), fruit (12%) | Cluster 2 |
| 3-Caffeoylquinic acid (mg/j) | Coffee (81%), fruit (11%) | Cluster 4 |
| 4-Caffeoylquinic acid (mg/j) | Coffee (87%) | Cluster 4 |
| 5-Caffeoylquinic acid (mg/j) | Coffee (64%), potatoes (15%), vegetables (10%) | Cluster 4 |
| 5-Feruloylquinic acid (mg/j) | Coffee (90%) | Cluster 4 |
| 4-Feruloylquinic acid (mg/j) | Coffee (81%) | Cluster 4 |
| 3-Feruloylquinic acid (mg/j) | Coffee (88%) | Cluster 4 |
| 3,5-Dicaffeoylquinic acid (mg/j) | Coffee (75%), vegetables (24%) | Cluster 4 |
| 3,4-Dicaffeoylquinic acid (mg/j) | Coffee (82%), vegetables (17%) | Cluster 4 |
| 4,5-Dicaffeoylquinic acid (mg/j) | Coffee (99%) | Cluster 4 |
| ***Other Polyphenols*** |  |  |
| Tyrosol (mg/j) | Wine (66%) | Cluster 2 |

Supplementary Table S3. Mean intakes of the main classes of polyphenols across clusters of dietary polyphenols identified in the SU.VI.MAX study (*n* 6092)

|  |  | Cluster 1 | | Cluster 2 | | Cluster 3 | | Cluster 4 | |  |
| --- | --- | --- | --- | --- | --- | --- | --- | --- | --- | --- |
|  |  | Mean | SD | Mean | SD | Mean | SD | Mean | SD | P Value |
| N |  | 1352 |  | 1355 |  | 1456 |  | 1929 |  |  |
| Total polyphenols (proanthocyanidins by RP-HPLC) (mg/d) | | 878.96 | 375.22 | 988.17 | 253.77 | 702.46 | 292.35 | 1440.84 | 446.10 | <0.0001 |
| Total polyphenols (proanthocyanidins by DP-HPLC) (mg/d) | | 1008.16 | 383.20 | 1114.81 | 264.43 | 878.54 | 349.49 | 1583.53 | 468.76 | <0.0001 |
| Sum of flavonoids (proanthocyanidins by RP-HPLC) (mg/d) | | 500.38 | 240.74 | 388.64 | 193.59 | 308.88 | 219.95 | 292.12 | 151.66 | <0.0001 |
| Sum of flavonoids (proanthocyanidins by DP-HPLC) (mg/d) | | 629.58 | 252.08 | 515.28 | 206.85 | 484.97 | 296.54 | 434.82 | 194.18 | <0.0001 |
|  | Proanthocyanidins, by DP-HPLC (mg/d) | 238.14 | 122.83 | 222.00 | 108.64 | 221.52 | 200.36 | 214.54 | 127.28 | <0.0001 |
|  | Proanthocyanidins, individual compounds (mg/d) | 108.95 | 49.82 | 95.37 | 48.02 | 45.43 | 34.60 | 71.84 | 50.23 | <0.0001 |
|  | Anthocyanins (mg/d) | 71.30 | 43.48 | 68.70 | 44.85 | 36.19 | 43.32 | 57.08 | 45.87 | <0.0001 |
|  | Chalcones (mg/d) | 1.06 10^-3^ | 4.29 10^-3^ | 1.02 10^-3^ | 3.51 10^-3^ | 5.28 10^-4^ | 3.27 10^-3^ | 9.78 10^-4^ | 3.24 10^-3^ | <0.0001 |
|  | Dihydrochalcones (mg/d) | 3.39 | 3.01 | 3.31 | 3.13 | 3.77 | 3.90 | 3.57 | 3.52 | 0.002 |
|  | Dihydroflavonols (mg/d) | 9.25 | 6.76 | 9.53 | 7.10 | 0.33 | 2.42 | 6.76 | 7.67 | <0.0001 |
|  | Theaflavins (mg/d) | 23.31 | 26.10 | 10.75 | 19.01 | 15.22 | 23.53 | 4.12 | 10.68 | <0.0001 |
|  | Catechins (mg/d) | 168.12 | 144.44 | 97.61 | 106.42 | 106.61 | 131.41 | 55.20 | 61.83 | <0.0001 |
|  | Flavanones (mg/d) | 26.79 | 28.00 | 26.42 | 27.54 | 26.19 | 31.87 | 24.71 | 27.73 | 0.16 |
|  | Flavones (mg/d) | 31.91 | 13.32 | 30.51 | 12.03 | 33.95 | 16.70 | 31.33 | 14.08 | <0.0001 |
|  | Flavonols (mg/d) | 62.90 | 29.86 | 51.14 | 25.02 | 46.92 | 30.01 | 42.33 | 22.26 | <0.0001 |
|  | Isoflavonoids (mg/d) | 0.02 | 0.47 | 0.01 | 0.02 | 0.01 | 0.20 | 0.01 | 0.02 | 0.14 |
| Sum of phenolic acids (mg/d) | | 342.32 | 241.12 | 561.02 | 108.53 | 369.23 | 187.14 | 1107.64 | 396.69 | <0.0001 |
|  | Hydroxybenzoic acids (mg/d) | 62.18 | 45.12 | 42.87 | 36.42 | 37.18 | 41.37 | 27.72 | 24.85 | <0.0001 |
|  | Hydroxycinnamic acids (mg/d) | 279.33 | 235.89 | 517.30 | 102.23 | 331.54 | 189.03 | 1079.19 | 395.68 | <0.0001 |
|  | Sum of other polyphenols (mg/d) | 29.73 | 17.17 | 31.84 | 16.34 | 23.66 | 19.65 | 36.24 | 18.24 | <0.0001 |
| Stilbenes (mg/d) | | 6.02 | 4.27 | 6.19 | 4.47 | 0.36 | 1.56 | 4.43 | 4.86 | <0.0001 |
| Lignans (mg/d) | | 0.51 | 0.15 | 0.48 | 0.15 | 0.33 | 0.12 | 0.40 | 0.15 | <0.0001 |
| Other phenolic acids (mg/d) | | 0.81 | 0.87 | 0.85 | 0.88 | 0.51 | 0.98 | 0.74 | 1.06 | <0.0001 |
